# A Next-Generation Sequencing Approach to Identify Gene Mutations in Early- and Late-Onset Hypertrophic Cardiomyopathy Patients of an Italian Cohort

**DOI:** 10.3390/ijms17081239

**Published:** 2016-07-30

**Authors:** Speranza Rubattu, Cristina Bozzao, Ermelinda Pennacchini, Erika Pagannone, Beatrice Maria Musumeci, Maria Piane, Aldo Germani, Camilla Savio, Pietro Francia, Massimo Volpe, Camillo Autore, Luciana Chessa

**Affiliations:** 1 Department of Clinical and Molecular Medicine, School of Medicine and Psychology, University Sapienza of Rome, 00185 Rome, Italy; cristina.bozzao@libero.it (C.B.); ariannaermelinda@hotmail.it (E.Pe.); epagannone@gmail.com (E.Pa.); beatrice.musumeci@uniroma1.it (B.M.M.); maria.piane@uniroma1.it (M.P.); aldo.germani@uniroma1.it (A.G.); camilla.savio@ospedalesantandrea.it (C.S.); pietro.francia@uniroma1.it (P.F.); massimo.volpe@uniroma1.it (M.V.); luciana.chessa@uniroma1.it (L.C.); 2 Department of Angiocardioneurology, IRCCS Neuromed, 86077 Pozzilli, Italy

**Keywords:** genetics, gene variants, hypertrophic cardiomyopathy, next-generation sequencing

## Abstract

Sequencing of sarcomere protein genes in patients fulfilling the clinical diagnostic criteria for hypertrophic cardiomyopathy (HCM) identifies a disease-causing mutation in 35% to 60% of cases. Age at diagnosis and family history may increase the yield of mutations screening. In order to assess whether Next-Generation Sequencing (NGS) may fulfil the molecular diagnostic needs in HCM, we included 17 HCM-related genes in a sequencing panel run on PGM IonTorrent. We selected 70 HCM patients, 35 with early (≤25 years) and 35 with late (≥65 years) diagnosis of disease onset. All samples had a 98.6% average of target regions, with coverage higher than 20× (mean coverage 620×). We identified 41 different mutations (seven of them novel) in nine genes: *MYBPC3* (17/41 = 41%); *MYH7* (10/41 = 24%); *TNNT2*, *CAV3* and *MYH6* (3/41 = 7.5% each); *TNNI3* (2/41 = 5%); *GLA*, *MYL2*, and *MYL3* (1/41=2.5% each). Mutation detection rate was 30/35 (85.7%) in early-onset and 8/35 (22.9%) in late-onset HCM patients, respectively (*p* < 0.0001). The overall detection rate for patients with positive family history was 84%, and 90.5% in patients with early disease onset. In our study NGS revealed higher mutations yield in patients with early onset and with a family history of HCM. Appropriate patient selection can increase the yield of genetic testing and make diagnostic testing cost-effective.

## 1. Introduction

Hypertrophic cardiomyopathy (HCM) is a common genetic cardiac disease that affects one out of 500 individuals from the general population [1]. It is a clinically variable and genetically heterogeneous disease. In fact, more than 20 genes were related with HCM and a total number of about 1400 distinct mutations were identified in affected patients [2]. The most frequently encountered mutations fall within myosin heavy chain 7 (*MYH7*) and myosin binding protein C (*MBPC3*) [3,4]. Sequencing of sarcomere protein genes in patients fulfilling clinical diagnostic criteria identifies a disease-causing mutation in only 35% to 60% of cases [5,6,7,8]. Identification of an HCM-causing mutation is an important step in the disease’s clinical management, not only to better support the clinical diagnosis in the proband but also to either exclude or confirm the presence of disease-causing mutations in other family members.

Considering the extreme genetic heterogeneity of the disease and the cost of genetic testing, several attempts were made to identify the clinical predictors of an underlying mutation [9,10,11]. In a large study of HCM patients genotyped for mutations in nine genes, the presence of a set of five clinical markers, including age at diagnosis <45 years, accounted for an 80% likelihood of positive genetic testing [11].

In addition, more reliable, precise, and possibly not time-consuming molecular diagnostic approaches are needed. In this regard, Next-Generation Sequencing (NGS), which has already been applied for the diagnosis of hereditary cardiovascular conditions as well as of other diseases [12,13,14,15,16], may represent a suitable tool. Targeted gene panels were shown to generate results with analytical quality identical to Sanger sequencing, and to have the advantage of being faster and cheaper with better coverage and sensitivity than that used in more expanded analyses.

The purpose of the present study was to analyse the yield of NGS applied to the genetic screening of a well-phenotyped Italian HCM cohort, composed of patients with both early- and late-onset diagnosis, also including patients with positive family history, and to explore the ability of NGS to accomplish the molecular diagnostic needs in clinical practice.

## 2. Results

### 2.1. Description of Study Population

The clinical characteristics of patients enrolled in the study are shown in Table 1A.

The patients were divided into two subgroups of 35 patients each, depending on the age at HCM diagnosis: the early-onset (EO) group with a mean age at diagnosis of 18.6 ± 8.5 years and the late-onset (LO) group with a mean age at diagnosis of 70.4 ± 4.8 years. The number of patients with a positive family history for HCM was significantly higher in the EO group (*p* = 0.0001) (Table 1B). Thirty-four patients were women and 36 were men. The sex distribution of patients was different in the two subgroups, with more males in the EO group (*p* = 0.0001). The left atrium size was significantly different in the two groups (*p* = 0.0001), with LO patients more frequently exhibiting left atrial enlargement. The obstructive form of HCM was less frequently observed in the EO as compared to the LO group (*p* = 0.03). Evolution of the disease towards end stage (left ventricular ejection fraction <50%) was observed only in the EO group. None of the other clinical features considered in the study was significantly different between the two groups.

### 2.2. Sequencing

The coding region of each of the 17 HCM phenotype causative genes included in the HCM panel was sequenced on Personal Genome Machine (PGM) IonTorrent sequencer. The 17 genes included in the HCM panel used for this analysis are shown in Table 2. Sequencing produced an average of 240,000 reads per patients; the mean read length was 130 bp; the average read depth per sample was 620× with a mean percentage of reads on target of 93.77%; the mean percentage of regions of interest (ROI) covered at least by 20× was 98.6%, and that covered at least by 100× was 94.7%. Details of the sequencing metrics for each patient are reported in Table 3.

Two hundred eighty-two variants were identified within the 17 genes analysed: two were ins/del, 175 were intronic, 37 missense, 59 synonimous, five splicing, and four stop mutations. After filtration, 41 variants with a possible clinical effect were selected and confirmed by Sanger sequencing (data not shown). These variants were located in nine of the 17 genes: *MYBPC3* (17/41 = 41%); *MYH7* (10/41 = 24%); troponin T2 (*TNNT2*), caveolin 3 (*CAV3*), and myosin heavy chain 6 (*MYH6*) (3/41 = 7.5% each); troponin I 3 (*TNNI3*) (2/41 = 4.8%); and galactosidase alpha (*GLA*), myosin light chain 2 (*MYL2*), and myosin light chain 3 (*MYL3*) (1/41 = 2.5% each). Thirty-four were known variants, whereas seven were novel. Out of the seven new missense mutations, four had uncertain significance, two were likely pathogenic, and one was likely benign. Considering the 34 known variants, 15 were known to have pathogenic effect, six were likely pathogenic, one was likely benign, and 12 were known registered variants but with unknown clinical significance (Table 4). Mutations in sarcomeric genes accounted for 90% of all identified mutations, with *MYBPC3* and *MYH7* alone accounting for 65% of all mutations. Considering only mutations in *MYBPC3*, eight missense mutations and nine truncating mutations were identified (Table 4).

### 2.3. Group Comparison after Sequencing

The mutation detection rate was 85.7% (30/35) in the EO group and 22.9% (8/35) in the LO group.

The number of patients in which the molecular screening allowed the identification of at least one mutation was significantly different in the two groups of patients with different age at diagnosis (*p* < 0.0001). The overall detection rate, regardless of the age of onset, was 54.3% (38/70). The NGS analysis confirmed the known mutational status of the 22 controls (seven positive and 15 negative) included in this study. Mutations identified in each patient are listed in Table 5. Considering only patients with positive family history, the detection rate was 88% (22/25), ranging from 75% (3/4) in the LO group to 90.5% (19/21) in the EO group. Considering sporadic cases only, the overall detection rate was 35.5%, with a significant difference between EO (11/14, 78.6%) and LO (5/31, 16%), *p* < 0.0002.

In the EO group, patients EO13 and EO33 carried three different mutations in *MYBPC3*. One of them was clinically characterized by an unfavourable course with evolution to end stage disease. 

Four patients carried two different mutations: EO23 carried two mutations in *MYH7*, whereas EO6, EO11, and EO21 carried two mutations in two different genes (Table 5). In the LO group, only two patients, LO8 and LO17, harboured two mutations in different genes (Table 5).

The distribution of the identified gene mutations was similar between the two groups with the exceptions of *MYH6* and *TNNT2*. In fact, mutations in *MYH6* were identified in the LO group only, whereas mutations in *TNNT2* were identified in the EO group only.

## 3. Discussion

This report describes the results of a genetic screening obtained through NGS approach in an Italian population of unrelated and clinically well characterized HCM cases, divided into two groups according to age at diagnosis. Our population included a good percentage of patients with a family history of HCM. As expected, the prevalence of familial forms was higher in the EO group, whereas the prevalence of sporadic forms was higher in the LO group.

The key finding of our investigation was the higher yield of mutation detection rate in the EO group and in patients with a family history of disease, with 90.5% of cases carrying an identified mutation. The overall yield of genetic testing was close to 50%, and, as previously reported in the literature [4,7,8,9,11], mutations in *MYBPC3* and *MYH7* accounted for about 65% of all variants. Other mutations were found in six additional sarcomeric genes (*TNNT2*, *CAV3*, *MYH6*, *TNNI3*, *MYL2*, and *MYL3*) and in one non-sarcomeric gene (*GLA*). Approximately a quarter of all variants were novel, most of them belonging to *MYH7*. The pathogenicity of novel mutations was verified through appropriate software for analysis.

HCM is a disease characterized by a relevant heterogeneity of both morphological and clinical features. For this reason, despite the growing knowledge on its genetic basis, the establishment of a more precise genotype–phenotype correlation has been difficult to achieve.

The main original aspect of our investigation was to test through NGS a wide range of HCM-causing genes (14 sarcomeric and three non-sarcomeric) while comparing the extreme ages of disease onset and evaluating the impact of familial occurrence of the disease even in patients with late diagnosis. Due to the small sample size of the population, our study could not address the issue of a relationship between genetic variants and phenotypic characteristics of different HCM onset patients. Notably, the presence of double and triple mutations was detected mostly among younger patients, and one of them showed a more severe form of the disease.

The different rate of pathogenic mutations found in HCM patients with early and late onset of the disease was consistent with the literature [17,18,19], confirming that some mutations can be found mainly in young HCM patients (*TNNT2*) whereas other mutations are detected exclusively in the elderly (*MYH6*) [17,18,19].

In our study, a majority of patients with young age at diagnosis had a positive genetic testing (80% of cases), four-fold higher than that of the elderly and sporadic HCM cases. These data, together with previous observations, reinforce the concept that age at HCM diagnosis is a powerful predictor of positive genetic testing [11,17,18,19]. We also support the notion that family history of HCM has a key role in appropriately addressing the genetic test. In fact, among HCM patients with a late diagnosis, those with a family history of the disease had a higher rate of mutation detection (75%).

We used an expanded panel of 17 genes in the attempt to improve the mutation detection rate. With this approach we mostly confirmed the type of mutations and the mutation distribution already described in the literature for HCM. In particular, the most frequent sarcomeric gene mutations, namely those in *MYBPC3* and *MYH7*, accounted for the majority of the positive findings. Moreover, six of the seven novel mutations identified in our patients were in the main sarcomeric genes (three in *MYH7*, one in *MYH6*, one in *MYBPC3*, and one in *TNNI3*). In this regard, the limitations of using a wide diagnostic panel for HCM genetic testing have been recently highlighted in one of the largest clinical genetic studies ever reported for HCM [20]. Consistently, a panel designed only for the main HCM genes (*n* = 9), was able to successfully screen a large cohort of HCM patients [21]. Our findings support the choice of a limited, well-selected panel of HCM genes as the best tool for diagnostic purposes.

## 4. Materials and Methods

### 4.1. Patient Selection

Seventy patients with clinical diagnosis of HCM were included in the study. We selected 35 patients with early diagnosis of the disease (≤25 years, EO-early onset) and 35 patients with a late diagnosis (≥65 years, LO-late onset). All patients underwent a cardiologic evaluation as well as genetic counselling. Clinical data for each patient included a detailed personal and family history and a thorough scrutiny of the age at which HCM was first diagnosed. Both electrocardiographic and echocardiographic examinations were performed at the time of inclusion into the study. The echocardiographic parameters included both structural measurements and resting LV outflow tract gradients derived from the continuous-wave Doppler velocities. The clinical diagnosis of HCM was based on the echocardiographic demonstration of a hypertrophied and not dilated left ventricle (wall thickness >15 mm in adults, or the equivalent wall thickness relative to body surface area in children) in the absence of another cardiac or systemic disease that could produce comparable left ventricular hypertrophy [22,23].

The mutational status for *MYH7*, *MYBPC3*, *TNNI3*, *TNNT2*, *TPM1*, and *MYL2* genes was already known in 22/70 patients (8 EO and 14 LO patients). All coding exons (±20 bp) of the six genes were previously analysed by Sanger sequencing. The 22 samples were included in our study as positive and negative controls for the six genes also present in our NGS panel. The seven positive controls carried mutations in *MYBPC3* (EO7, EO29, EO33, EO35), *MYH7* (LO13), *TNNI3* (EO30), and *MYL2* (EO20). The 15 negative controls for the six genes were: EO10, EO15, LO5, LO6, LO9, LO12, LO19, LO21, LO22, LO25, LO27, LO28, LO29, LO32, and LO33. 

This study was conducted in accordance with the ethical standards of the institutional and/or national research committee and with the 1964 Helsinki Declaration and its later amendments or comparable ethical standards (The approval identification number: 42 of 28 September 2007). A signed informed consent for blood sampling was obtained from all patients included in the study.

### 4.2. DNA Extraction and Quantification

Genomic DNA was extracted from peripheral whole blood using a commercially available kit (Invitrogen, Milan, Italy), and then quantified using Qubitds DNA HS Assay Kit on Qubit 2.0 Fluorometer (Invitrogen).

### 4.3. Sequencing

Seventeen genes known to be causative of HCM phenotype were selected for targeted sequencing (Table 2). A custom panel for coding DNA (+/−25 bp of intronic flanking regions) analysis of selected genes was designed online using Ion AmpliSeq Designer 2.0.3 (https://www.ampliseq.com/browse.action) [24]. The final custom panel was composed of 358 amplicons divided into two primer pools for a total of 61.89 kb of DNA. The panel covered 96.47% of regions of interest (ROI). Libraries were prepared using Ion AmpliSeq Library Kit v2.0 (Life Technologies, Carlsbad, CA, USA), according to the manufacturer’s instructions. One of 16 barcodes of the Ion Xpress Barcode Adapters1-16 Kit (Thermo Fisher Scientific Life Sciences Solutions, Carlsbad, CA, USA) was added to each sample. Libraries were quantified with Qubit dsDNA HS Assay Kit on Qubit 2.0 Fluorometer (Molecular Probes, Eugene, OR, USA) and equimolar amounts of each library were used to prepare template for clonal amplification. Emulsion PCR with Ion PGM Template OT2 200 Kit (Life Technologies, Carlsbad, CA, USA) was performed on OneTouch2 Systems (Life Technologies, Carlsbad, CA, USA). Templates were enriched using Ion OneTouch ES (Life Technologies, Carlsbad, CA, USA) and prepared for 316v2 chip loading (Life Technologies, Carlsbad, CA, USA). Groups from 12 to 16 sample libraries were sequenced on each chip. Sequencing runs were performed on Ion Torrent Personal Genome Machine (PGM, Life Technologies) using Ion PGM Sequencing 200 Kit v2, according to the manufacturer’s instructions.

### 4.4. Alignment

Data analysis was performed using the Torrent Suite Software v.4.0.2. (Life Technologies, Carlsbad, CA, USA). Reads were aligned to human reference genome hg19 from UCSC Genome Browser [25] and to a designed bed file from Ion AmpliSeq Designer results. Alignments were visually verified with Integrative Genomics Viewer IGV v.2.3, Broad Institute [26].

### 4.5. Coverage Analysis

The average read depth and the percentage of reads that mapped on ROI out of the total number of reads (reads on target) was calculated using Coverage Analysis plug-in (Life Technologies, Carlsbad, CA, USA). For each sample the percentage of ROI covered by at least 100× and 20× using amplicon coverage matrix file was calculated.

### 4.6. Variant Analysis

Variant calling was performed with Variant Caller plug-in configured with germ line-low stringency parameters. Variants were annotated using Ion Reporter 4.0 software (Carlsbad, CA, USA) [27]. Common single nucleotide variants (minor allele frequency MAF>5%, source 1000 Genomes), exonic synonymous variants, and intronic variants were removed from the analysis, while exonic non-synonymous, splice-site, and loss-of-function variants were analysed. The novel variants were analysed by means of three types of prediction software (SIFT, POLYPHEN, and PROVEAN) and classified based on the concordance of the prediction between the three types: “likely pathogenic,” “likely benign” (3/3 concordance), or “uncertain significance” (2/3 concordance).

### 4.7. Variant Validation

The identified variants were validated by Sanger sequencing using standard protocols. Specific primers were designed for the analysis. Polymerase Chain Reaction (PCR) products were directly sequenced by using the BigDye Terminator v3.1 Cycle Sequencing Kit (Life Technologies Corporation, Carlsbad, CA, USA). Sample analysis was performed on an ABI PRISM 3130xl Genetic Analyser (Applied Biosystems, Carlsbad, CA, USA).

### 4.8. Statistical Analysis

Statistical analysis was performed with SPSS statistical software (SPSS Inc., Chicago, IL, USA, version 17.0). Continuous variables are expressed as mean±SD. Comparisons between the two groups were performed using a Student’s *t*-test. The association between the mutational status and the clinical features of the two patient groups was evaluated using Chi-square and Fisher’s exact tests. A *p* value was considered statistically significant when <0.05. 

## 5. Conclusions

In summary, through NGS, we were able to detect pathogenic mutations responsible for HCM, particularly in patients with early onset of the disease and in those with a family history of HCM. Our findings document the suitability of a novel molecular diagnostic strategy for clinical purposes and the important role of appropriate patient selection in making genetic molecular testing more cost-effective.

## Figures and Tables

**Table:** (**A**)

Variables	Early-Onset *n* = 36	Late-Onset *n* = 35	*p*
Age at diagnosis (years)	18.6 ± 8.5	70.4 ± 4.8	0.0001
Male	27 (77.1)	9 (25.7)	0.0001
LV obstruction	14 (40)	24 (68.6)	0.03
Family history of HCM	21 (60)	4 (11.4)	0.0001
NYHA functional class			
I	24 (68.6)	4 (11.4)	0.0001
II	9 (25.7)	25 (71.4)	
III	2 (5.7)	6 (17.1)	
Unexplained syncope	5 (14.3)	6 (17.1)	1
Non sustained ventricular tachycardia	6 (24)	5 (22.7)	1
Left atrial dimension (mm)	39.3 ± 6.2	45 ± 4.5	0.0001
Maximal LV wall thickness (mm)	21.4 ± 6.2	18.7 ± 2.6	0.02
Late gadolinium enhancement	24/29 (82.8)	9/19 (47.4)	0.01
Atrial fibrillation	11 (31.4)	10 (28.6)	1
End stage disease	4 (11.4)	0 (0)	0.11
Myectomy	2 (5.7)	0 (0)	0.49
ICD implantation	12 (34.3)	2 (5.7)	0.006
Death	0 (0)	1 (2.9)	1

**Table:** (**B**)

Patients	All *n* = 70	EO *n* = 35	LO *n* = 35	*p*
Familial HCM	25 (36)	21 (60)	4 (14.4)	0.0001
Sporadic HCM	45 (64)	14 (40)	31 (88.6)	0.0001

In (**A**): Continuous variables are expressed as mean ± SD. Qualitative variable are expressed as *n* (%). HCM: hypertrophic cardiomyopathy; NYHA: New York Functional Class; LV: left ventricular; ICD: implantable cardioverter defibrillator; In (**B**): Variable are expressed as *n* (%); EO: early-onset; LO: late-onset.

**Table 2:** Metrics of the 17 genes included into the HCM panel.

#No.	Gene Name	Ref Seq NCBI	Genomic Location (hg19)	Description	Amplicons	Coverage (%)	Target (bp)	Missed (bp)
1	*MYBPC3*	NM_000256	chr11:47352958-47374253	myosin binding protein C, cardiac	53	100	5458	105
2	*MYH7*	NM_000257	chr14:23881948-23904870	myosin, heavy chain 7, cardiac muscle, β	67	98	7746	231
3	*TPM1*	NM_001018005	chr15:63334838-63364111	tropomyosin 1 α chain isoform 7	23	99.91	2245	2
4	*TNNT2*	NM_001001430	chr1:201328143-201346805	troponin T type 2, cardiac isoform 1	20	100	2357	0
5	*TNNI3*	NM_000363	chr19:55663137-55669100	troponin I, cardiac	10	99.9	989	1
6	*MYL2*	NM_000432	chr12:111348626-111358404	slow cardiac myosin regulatory light chain 2	9	84.8	858	46
7	*MYL3*	NM_000258	chr3:46899357-46904973	slow skeletal ventricular myosin alkali light	9	94,6	894	136
8	*ACTC1*	NM_005159	chr15:35080297-35087927	cardiac muscle α actin 1 proprotein	13	100	1440	0
9	*LAMP2*	NM_002294	chrX:119560004-119603204	lysosomal-associated membrane protein 2 isoform	21	100	2077	0
10	*PRKAG2*	NM_016203	chr7:151253203-151574316	AMP-activated protein kinase γ 2 subunit	26	84.3	2713	426
11	*GLA*	NM_000169	chrX:100652779-100663001	α-galactosidase A precursor	14	100	1647	0
12	*MYH6*	NM_002471	chr14:23851199-23877482	myosin heavy chain 6	66	94.52	7707	422
13	*TNNC1*	NM_003280	chr3:52485108-52488057	troponin C, slow	8	98.2	792	14
14	*CSRP3*	NM_003476	chr11:19203578-19223589	cysteine and glycine-rich protein 3	8	100	840	0
15	*PLN*	NM_002667	chr6:118869442-118881586	phospholamban	2	100	210	0
16	*TCAP*	NM_003673	chr17:37821599-37822806	telethonin	5	100	606	0
17	*CAV3*	NM_033337	chr3:8775486-8788451	Homo sapiens caveolin 3 (CAV3), transcript variant 1, mRNA.	4	100	558	0

Gene symbols: *TPM1*: tropomyosin 1; *ACTC1*: actin, α, cardiac muscle 1; *LAMP2*: lysosomal associated membrane protein 2; *PRKAG2*: protein kinase AMP-activated non-catalytic subunit γ 2; *TNNC1*: troponin C 1; *CSRP3*: cystein and glycine-rich protein 3; *PLN*: phospholamban; *TCAP*: telethonin.

**Table 3:** Patient sequencing metrics.

Patients	Mapped Reads	Reads on Target (%)	Uniformity (%)	ROI MEAN COVERAGE	ROI ≥ 20× (%)	*n* of Amplicons < 20×	ROI ≥ 100× (%)	*n* of Amplicons < 100×
EO1	178,727	92.13	93.95	459.94	98.60	5	94.97	18
EO2	178,731	90.57	94.83	452.19	98.88	4	95.53	16
EO3	72,440	91.78	93.75	185.71	96.93	11	83.52	59
EO4	247,711	90.70	93.90	627.61	99.44	2	96.09	14
EO5	111,232	91.03	93.57	282.82	97.21	10	91.62	30
EO6	280,419	93.08	94.15	729.08	99.16	3	96.09	14
EO7	623,594	92.53	93.81	1611.77	99.44	2	98.32	6
EO8	561,715	97.46	92.18	1529.12	99.44	2	97.49	9
EO9	77,846	93.36	93.83	203.00	96.93	11	86.87	47
EO10	381,796	96.33	93.71	1027.32	99.44	2	97.21	10
EO11	311,658	93.28	93.70	812.08	99.44	2	96.09	14
EO12	239,783	93.00	94.15	622.93	98.88	4	95.53	16
EO13	276,453	93.48	94.44	721.90	99.44	2	96.09	14
EO14	215,672	93.30	94.53	562.09	99.44	2	95.81	15
EO15	465,323	94.73	93.01	1231.34	99.44	2	96.65	12
EO16	465,619	97.25	92.65	1264.84	99.44	2	96.93	11
EO17	441,220	95.42	92.96	1176.05	99.72	1	97.49	9
EO18	192,373	98.07	91.50	526.97	98.60	5	94.13	21
EO19	313,968	95.80	93.72	840.19	99.16	3	96.65	12
EO20	192,211	95.35	94.02	517.24	98.60	5	95.81	15
EO21	196,251	95.05	94.02	521.07	98.88	4	95.81	15
EO22	303,435	96.01	93.55	813.79	98.88	4	96.65	12
EO23	322,467	94.14	92.17	847.94	98.88	4	95.81	15
EO24	253,552	95.97	91.35	679.71	98.88	4	95.53	16
EO25	188,696	95.33	93.96	502.45	98.60	5	95.53	16
EO26	182,956	94.99	92.88	485.47	98.88	4	94.13	21
EO27	191,880	94.62	93.58	507.12	98.88	4	94.97	18
EO28	228,313	92.89	93.22	592.43	98.88	4	94.69	19
EO29	199,442	98.07	92.15	546.33	98.32	6	94.97	18
EO30	190,915	97.24	92.66	518.58	98.04	7	94.69	19
EO31	161,793	95.49	92.19	431.54	97.77	8	93.30	24
EO32	245,414	89.57	93.45	613.99	98.32	6	95.81	15
EO33	205,079	95.46	85.54	546.83	96.65	12	89.39	38
EO34	210,900	97.24	93.66	572.83	98.60	5	95.25	17
EO35	147,306	97.24	92.62	402.14	98.32	6	94.13	21
LO1	178,290	90.77	93.68	321.94	97.77	8	91.90	29
LO2	205,008	93.84	93.97	537.36	99.16	3	96.09	14
LO3	159,828	93.15	93.80	502.12	98.88	4	95.53	16
LO4	193,973	93.90	94.09	1062.97	99.44	2	96.37	13
LO5	191,160	93.72	93.21	1097.36	99.16	3	96.65	12
LO6	177,316	94.10	93.42	931.78	99.44	2	96.37	13
LO7	238,812	94.23	93.77	593.70	97.77	8	92.18	28
LO8	158,483	93.67	93.01	708.10	99.44	2	97.49	9
LO9	213,370	93.89	94.34	861.05	99.44	2	97.21	10
LO10	190,285	94.47	93.70	415.86	98.32	6	94.41	20
LO11	182,160	93.99	94.02	505.35	98.32	6	94.97	18
LO12	213,052	92.51	94.69	532.23	98.88	4	94.97	18
LO13	249,591	93.48	93.92	304.45	96.93	11	90.78	33
LO14	195,422	94.82	94.22	378.77	98.04	7	92.74	26
LO15	201,815	95.14	93.81	717.45	98.60	5	95.53	16
LO16	400,156	98.18	90.79	500.41	98.60	5	94.41	20
LO17	274,868	95.75	91.07	423.42	98.04	7	92.74	26
LO18	158,695	92.07	94.09	457.87	98.32	6	94.97	18
LO19	195,752	89.66	93.66	206.68	96.65	12	86.03	50
LO20	83,846	88.25	93.28	490.24	98.32	6	94.13	21
LO21	179,015	91.57	94.00	408.12	98.60	5	94.69	19
LO22	170,180	89.07	93.89	735.17	98.60	5	94.97	18
LO23	161,290	90.82	93.26	680.84	99.16	3	96.09	14
LO24	281,769	92.16	93.07	536.34	98.60	5	95.53	16
LO25	145,219	93.37	93.76	517.60	99.16	3	95.81	15
LO26	390,025	97.57	92.65	508.78	98.88	4	95.53	16
LO27	124,848	87.30	93.29	651.69	99.16	3	95.25	17
LO28	214,031	89.02	93.84	550.53	99.16	3	96.37	13
LO29	203,428	88.93	94.12	479.56	98.88	4	95.53	16
LO30	127,496	90.40	93.91	452.05	98.32	6	94.69	19
LO31	25,524	95.49	93.62	409.17	98.04	7	93.02	25
LO32	329,472	93.56	94.58	559.60	99.16	3	96.09	14
LO33	265,155	95.60	93.94	414.68	98.32	6	94.13	21
LO34	21,736	97.78	89.25	628.59	98.88	4	95.81	15
LO35	352,805	94.55	92.69	466.05	97.77	8	94.69	19

**Table 4:** Mutations detected per gene.

Gene ID	Chrom	Position	Exon	DNA Change	Protein Change	Mutation Type	dbSNP	Prev. Rep.	GMAF	SIFT	POLYPHEN	PROVEAN (cutoff = −2.5)	Clinical Significance
*CAV3*	chr3	8787313	2	c.216C>G	Cys72Trp	MISSENSE	rs116840776	yes	0.00100 (G)	deleterious 0	probably damaging 0.999	deleterious −6.167	known/uncertain significance
	chr3	8787330	2	c.233C>T	Thr78Met	MISSENSE	rs72546668	yes	0.00200 (T)	tolerated 0.05	possibly damaging 0.537	neutral −0.833	known/uncertain significance
	chr3	8787497	2	c.400G>T	Ala134Ser	MISSENSE				deleterious 0.01	benign 0.07	neutral 0.862	new/uncertain significance
*GLA*	chrX	100653420	6	c.937G>T	Asp313Tyr	MISSENSE	rs28935490	yes	0.0021 (A)	deleterious 0	probably damaging 0.952	deleterious −3.183	known/uncertain significance
*MYBPC3*	chr11	47371426	5	c.553A>T	Lys185Ter	STOP	rs375607980	yes					known/pathogenic
	chr11	47371414	5	c.565G>A	Val189Ile	MISSENSE	rs11570052	yes	0.00200 (T)	tolerated 0.44	benign 0.132	Neutral −0.418	known/likely benign
	chr11	47365154	13	c.1112C>G	Pro371Arg	MISSENSE	rs397515887	yes	0.00020 (A)	deleterious 0	probably damaging 0.994	deleterious −8.043	known/uncertain significance
	chr11	47365147	13	c.1120C>T	Gln374Ter	STOP	rs730880635	yes					known/pathogenic
	chr11	47364429	15	c.1409G>A	Arg470Gln	MISSENSE		yes		deleterious 0.01	probably damaging 0.982	deleterious −3.094	known/uncertain significance
	chr11	47364270	16	c.1483C>T	Arg495Trp	MISSENSE	rs397515905	yes		deleterious 0	probably damaging 0.999	deleterious −5.228	known/uncertain significance
	chr11	47364162	16	c.1591G>C	Gly531Arg	MISSENSE	rs397515912	yes	0.00020 (G)	deleterious 0	probably damaging 0.996	deleterious −7.038	known/likely pathogenic
	chr11	47364129	16	c.1624G>C	Glu542Gln	MISSENSE/SPLICING	rs121909374	yes	0.00008 (G)				known/pathogenic
	chr11	47360071	22	c.2308G>A	Asp770Asn	MISSENSE/SPLICING	rs36211723	yes					known/pathogenic
	chr11	47359347	23	c.2309-2A>G		SPLICING	rs111729952	yes					known/pathogenic
	chr11	47359115	24	c.2429G>A	Arg810His	MISSENSE	rs375675796	yes	0.00008 (T)	deleterious 0	probably damaging 1	deleterious −4.564	known/likely pathogenic
	chr11	47359085	24	c.2459G>A	Arg820Gln	MISSENSE	rs2856655	yes		deleterious 0	probably damaging 0.98	deleterious −2.925	known/likely pathogenic
	chr11	47356592	26	c.2905+1G>A		SPLICING	rs397515991	yes					known/pathogenic
	chr11	47355264	28	c.3034C>T	Gln1012Ter	STOP	rs730880586	yes					known/pathogenic
	chr11	47354882	29	c.3192_3193insC	Lys1065Glnfs	INS	rs397516007	yes					known/pathogenic
	chr11	47353801	32	c.3636T>G	Ile1212Met	MISSENSE				deleterious 0	probably damaging 0.918	deleterious −2.498	new/likely pathogenic
	chr11	47353662	32	c.3775C>T	Gln1259Ter	STOP	rs730880605	yes					known/pathogenic
*MYH7*	chr14	23900850	8	c.676G>A	Ala226Thr	MISSENSE				deleterious 0	probably damaging 0.985	neutral −1.757	new/uncertain significance
	chr14	23896866	16	c.1816G>A	Val606Met	MISSENSE	rs121913627	yes					known/pathogenic
	chr14	23896042	18	c.1988G>A	Arg663His	MISSENSE	rs371898076	yes	0.00008 (T)				known/pathogenic
	chr14	23895189	19	c.2146G>C	Gly716Arg	MISSENSE	rs121913638	yes		deleterious 0.01	probably damaging 0.995	deleterious −3.728	known/likely pathogenic
	chr14	23895179	19	c.2156G>A	Arg719Gln	MISSENSE	rs121913641	yes					known/pathogenic
	chr14	23894116	22	c.2543_2545 delAAG	Lys847del	DEL		yes					known/pathogenic
	chr14	23893234	23	c.2804A>T	Glu935Val	MISSENSE	rs730880761	yes					known/pathogenic
	chr14	23891501	25	c.3133C>T	Arg1045Cys	MISSENSE	rs45611033	yes	0.00020 (A)	deleterious 0.03	benign 0.203	deleterious −6.180	known/uncertain significance
	chr14	23889413	27	c.3367G>C	Glu1123Gln	MISSENSE				deleterious 0.01	probably damaging 0.968	neutral −2.389	new/uncertain significance
	chr14	23887615	30	c.3973G>A	Ala1325Thr	MISSENSE/SPLICING				deleterious 0.02	possibly damaging 0.751	neutral −2.329	new/uncertain significance
*TNNT2*	chr1	201334751	9	c.281G>C	Arg94Thr	MISSENSE	rs397516452	yes		deleterious 0	possibly damaging 0.573	deleterious −5.588	known/uncertain significance
	chr1	201330414	14	c.794A>T	Lys265Ile	MISSENSE	rs397516482	yes		deleterious 0	probably damaging 0.958	deleterious −6.86	known/uncertain significance
	chr1	201328373	16	c.853C>T	Arg285Cys	MISSENSE	rs121964857	yes		tolerated 0.06	probably damaging 0.978	neutral −2.09	known/likely pathogenic
*MYH6*	chr14	23873951	7	c.611G>A	Arg204His	MISSENSE	rs200623022	yes		tolerated 0.05	possibly damaging 0.807	neutral −1.327	known/uncertain significance
	chr14	23865497	20	c.2425C>T	Arg809Cys	MISSENSE				deleterious 0	probably damaging 0.963	deleterious −5.294	new/likely pathogenic
	chr14	23853697	36	c.5519A>G	Lys1840Arg	MISSENSE	rs373629059			tolerated 0.13	probably damaging 0.999	neutral −1.731	known/uncertain significance
*MYL2*	chr12	111350901	6	c.401A>C	Glu134Ala	MISSENSE	rs143139258	yes		deleterious 0.01	possibly damaging 0.755	Deleterious −5.696	known/likely pathogenic
*MYL3*	chr3	46902303	3	c.170C>A	Ala57Asp	MISSENSE	rs139794067	yes		deleterious 0	probably damaging 0.996	deleterious −5.236	known/uncertain significance
*TNNI3*	chr19	55665561	6	c.385C>G	Thr128Ser	MISSENSE				tolerated 0.186	benign 0.000	neutral 0.61	new/likely benign
	chr19	55665516	6	c.431T>A	Leu144Gln	MISSENSE	rs121917760	yes					known/pathogenic

Prev. Rep.: previously reported; GMAF: Global minor allele frequency; Software prediction programs used for sequence variant interpretation: SIFT: Evolutionary conservation; POLYPHEN: Protein structure/function and evolutionary conservation; PROVEAN: Alignment and measurement of similarity between variant sequence and protein sequence homolog.

**Table 5:** Mutations detected per patient.

Early-Onset											
Patient ID	Familiarity	Gene ID	Exon	DNA Change	Protein Change	Mutation Type	Clinical Significance	dbSNP	Previously Reported	Coverage	Allele Coverage
EO1	yes	*MYBPC3*	5	c.553A>T	Lys185Ter	STOP	known/pathogenic	rs375607980	yes	384	202
EO2	yes	*MYH7*	19	c.2156G>A	Arg719Gln	MISSENSE	known/pathogenic	rs121913641	yes	399	204
EO3		*CAV3*	2	c.233C>T	Thr78Met	MISSENSE	known/uncertain significance	rs72546668	yes	124	57
EO4		*MYBPC3*	23	c.2309-2A>G		SPLICING	known/pathogenic	rs111729952	yes	400	186
EO5		*MYH7*	16	c.1816G>A	Val606Met	MISSENSE	known/pathogenic	rs121913627	yes	383	204
EO6	yes	*MYH7*	8	c.676G>A	Ala226Thr	MISSENSE	new/uncertain significance			399	208
		*GLA*	6	c.937G>T	Asp313Tyr	MISSENSE	known/uncertain significance	rs28935490	yes	399	183
EO7	yes	*MYBPC3*	28	c.3034C>T	Gln1012Ter	STOP	known/pathogenic	rs730880586	yes	397	194
EO8	yes	*MYBPC3*	23	c.2309-2A>G		SPLICING	known/pathogenic	rs111729952	yes	398	204
EO9	yes	*MYH7*	19	c.2146G>C	Gly716Arg	MISSENSE	known/likely pathogenic	rs121913638	yes	354	169
EO11	yes	*MYBPC3*	16	c.1483C>T	Arg495Trp	MISSENSE	known/uncertain significance	rs397515905	yes	400	259
		*CAV3*	2	c.216C>G	Cys72Trp	MISSENSE	known/uncertain significance	rs116840776	yes	400	182
EO12	yes	*MYBPC3*	23	c.2309-2A>G		SPLICING	known/pathogenic	rs111729952	yes	399	185
EO13		*MYBPC3*	32	c.3636T>G	Ile1212Met	MISSENSE	new/likely pathogenic			400	201
			23	c.2309-2A>G		SPLICING	known/pathogenic	rs111729952	yes	399	187
			16	c.1591G>C	Gly531Arg	MISSENSE	known/likely pathogenic	rs397515912	yes	400	184
EO14		*CAV3*	2	c.233C>T	Thr78Met	MISSENSE	known/uncertain significance	rs72546668	yes	399	214
EO17		*MYBPC3*	22	c.2308G>A	Asp770Asn	MISSENSE/SPLICING	known/pathogenic	rs36211723	yes	399	195
EO18	yes	*MYBPC3*	13	c.1120C>G	Tyr374Ter	STOP	known/pathogenic	rs730880635	yes	400	225
EO19	yes	*MYBPC3*	32	c.3775C>T	Gln1259Ter	STOP	known/pathogenic	rs730880605	yes	398	204
EO20	yes	*MYL2*	6	c.401A>C	Glu134Ala	MISSENSE	known/likely pathogenic	rs143139258	yes	398	191
EO21	yes	*MYBPC3*	5	c.565G>A	Val189Ile	MISSENSE	known/uncertain significance	rs11570052	yes	309	253
		*MYH7*	22	c.2543_2545 delAAG	Lys847del	DELETION	known/pathogenic		yes	391	194
EO22		*CAV3*	2	c.400G>T	Ala134Ser	MISSENSE	new/uncertain significance			330	168
EO23		*MYH7*	30	c.3973G>A	Ala1325Thr	MISSENSE/SPLICING	new/uncertain significance			400	176
			23	c.2804A>T	Glu935Val	MISSENSE	known/pathogenic	rs730880761	yes	400	206
EO25	yes	*MYBPC3*	15	c.1409G>A	Arg470Gln	MISSENSE	known/uncertain significance		yes	293	130
EO26	yes	*TNNT2*	16	c.853C>T	Arg285Cys	MISSENSE	known/likely pathogenic	rs121964857	yes	323	167
EO27	yes	*TNNT2*	14	c.794A>T	Lys265Ile	MISSENSE	known/uncertain significance	rs397516482	yes	395	193
EO29	yes	*MYBPC3*	24	c.2429G>A	Arg810His	MISSENSE	known/likely pathogenic	rs375675796	yes	400	148
EO30	yes	*TNNI3*	6	c.431T>A	Leu144Gln	MISSENSE	known/pathogenic	rs121917760	yes	398	227
EO31		*MYBPC3*	5	c.565G>A	Val189Ile	MISSENSE	known/likely benign	rs11570052	yes	312	152
EO32		*MYH7*	18	c.1988G>A	Arg663His	MISSENSE	known/pathogenic	rs371898076	yes	400	211
EO33		*MYBPC3*	29	c.3193_3194 insC	Lys1065Glnfs	INSERTION	known/pathogenic	rs397516007	yes	398	194
			16	c.1591G>C	Gly531Arg	MISSENSE	known/likely pathogenic	rs397515912	yes	400	212
			13	c.1112C>G	Pro371Arg	MISSENSE	known/uncertain significance	rs397515887	yes	235	87
EO34	yes	*TNNT2*	9	c.281G>C	Arg94Thr	MISSENSE	known/uncertain significance	rs397516452	yes	400	196
EO35		*MYBPC3*	26	c.2905+1G>A	ex26	SPLICING	known/pathogenic	rs397515991	Yes	296	139
**Late-Onset**											
**Patient ID**	**Familiarity**	**Gene ID**	**Exon**	**DNA Change**	**Protein Change**	**Mutation Type**	**Clinical Significance**	**dbSNP**	**Previously Reported**	**Coverage**	**Allele Coverage**
LO1	Yes	*MYH7*	25	c.3133C>T	Arg1045Cys	MISSENSE	known/uncertain significance	rs45611033	yes	213	113
LO4		*MYH7*	27	c.3367G>C	Glu1123Gln	MISSENSE	new/uncertain significance			400	220
LO6		*MYH6*	20	c.2425C>T	Arg809Cys	MISSENSE	new/ likely pathogenic			299	139
LO8	Yes	*MYBPC3*	16	c.1624G>C	Glu542Gln	MISSENSE/SPLICING	known/pathogenic	rs121909374	yes	353	188
		*TNNI3*	6	c.385C>G	Thr128Ser	MISSENSE	new/likely benign			400	204
LO13	Yes	*MYH7*	16	c.1816G>A	Val606Met	MISSENSE	known/pathogenic	rs121913627	yes	383	204
LO14		*MYH6*	36	c.5519A>G	Lys1840Arg	MISSENSE	known/uncertain significance	rs373629059	yes	399	196
LO16		*MYBPC3*	24	c.2459G>A	Arg820Gln	MISSENSE	known/likely pathogenic	rs2856655	yes	400	213
LO17		*MYH6*	7	c.611G>A	Arg204His	MISSENSE	known/uncertain significance	rs200623022	yes	398	201
		*MYL3*	3	c.170C>A	Ala57Asp	MISSENSE	known/uncertain significance	rs139794067	yes	398	168

dbSNP: database single nucleotide polymorphisms (www.ncbi.nlm.nih.gov/SNP).
